# A Survey of AI-Based Anomaly Detection in IoT and Sensor Networks

**DOI:** 10.3390/s23031352

**Published:** 2023-01-25

**Authors:** Kyle DeMedeiros, Abdeltawab Hendawi, Marco Alvarez

**Affiliations:** Department of Computer Science and Statistics, College of Arts and Sciences, University of Rhode Island, 1 Upper College Road, Kingston, RI 02881, USA

**Keywords:** sensors, IoT, anomaly detection, graphs, machine learning, neural networks

## Abstract

Machine learning (ML) and deep learning (DL), in particular, are common tools for anomaly detection (AD). With the rapid increase in the number of Internet-connected devices, the growing desire for Internet of Things (IoT) devices in the home, on our person, and in our vehicles, and the transition to smart infrastructure and the Industrial IoT (IIoT), anomaly detection in these devices is critical. This paper is a survey of anomaly detection in sensor networks/the IoT. This paper defines what an anomaly is and surveys multiple sources based on those definitions. The goal of this survey was to highlight how anomaly detection is being performed on the Internet of Things and sensor networks, identify anomaly detection approaches, and outlines gaps in the research in this domain.

## 1. Introduction

Machine learning (ML) and deep learning (DL), in particular, are common tools for anomaly detection [1,2,3,4,5,6]. With the rapid increase in the number of Internet-connected devices, the growing desire for Internet of Things (IoT) devices in the home, on our person, and in our vehicles, and the transition to smart infrastructure and the Industrial IoT (IIoT), anomaly detection in these devices is critical. This paper is a survey of anomaly detection in the IoT/sensor networks. This paper defines what an anomaly is and surveys multiple sources based on those definitions.

The following resources were searched:

- ACM Digital Library;
- arXiv;
- IEEE Xplore;
- Google Scholar.

The selection criteria focused primarily on the last few years of anomaly detection research (2019–2022). Papers were discarded from the final survey if they did not meet the criteria of performing anomaly detection or performed AD, but did not do so on sensor networks. Some of these papers are still referenced here for their usefulness in highlighting graph-based data representation, IoT use, or analyses of ML techniques.

Depending on the source, the definition of an anomaly varies. In [7], an anomaly was defined as “unexpected incidence significantly deviating from the normal patterns formed by the majority of the data-set”. In [8], an anomaly was defined as “a data object that deviates significantly from the majority of data objects”, while [9] defined an anomaly as “a mismatch between a node and its surrounding contexts”. In [2], anomalies were broken down into three types: point, contextual, and collective. In addition, [10] laid out eight types of anomalies using more specific, task-based definitions: denial of service, data type probing, malicious control, malicious operation, scan, spying (all of these are attack types), and wrong setup (non-attack).

In each of these cases, anomalies generally arise from the following causes:

- Malicious attack;
- Sensor fault;
- A significant environmental change, which is registered as an abnormal state by the sensor.

What causes each of these anomalies can be distinct, but it is not required to be so. A malicious attack can be performed to leverage the computing power of an IoT network, as seen in [11], or it can be performed to damage a system by producing false data, as seen in [12]. For the former, this could manifest as extra erroneous network traffic; for the latter, this can manifest as false messages being passed off as legitimate.

In addition, anomalies can be instantaneous or within some window of time. A rapid temperature change in a temperature sensor from one time step to another could indicate a severe environmental change or a sensor fault, but a gradual change over the course of hours or days could be normal operating behavior. For instance, in [13], NASA’s Turbofan data-set was used. As any motor runs, its temperature gradually changes. This would be nominal behavior, but a rapid increase in temperature or a temperature exceeding some threshold would be anomalous. How an approach handles the data over time can affect the success of the approach. There are specific approaches surveyed here, such as [14], which focus on anomaly detection in time series data.

In order to capture these different nuances for anomalies in the IoT and sensor networks, we define an anomaly in line with [8], while also partitioning anomalies into the following groups (similar to [2]):

- Point-based anomaly;
- Collective anomaly;
- Continuous anomaly.

A point-based anomaly is defined as a specific datum that contains anomalous information. A collective anomaly is a series of records (based on some window size) that contain anomalous data when compared to the remaining data sensed. A continuous anomaly is a collective anomaly with the window size approaching infinity from some starting point. Each paper in this survey focuses on specific types of anomalies; therefore, additional information on how a particular paper defines an anomaly will be given when appropriate.

This survey focuses on anomaly detection for sensor networks/the IoT. A sensor network is any collection of devices that sense information and report that information either to a centralized node or to other members of the network. The IoT is a network of (usually small) Internet-connected devices that perform some set of tasks. These devices may report their status to a central node or communicate with other devices connected to the network. Because the IoT and sensor networks perform largely the same job (to sense something, then report it), this survey treats these terms interchangeably. However, it should be noted that sensor networks are not strictly composed of IoT devices and IoT devices do not just sense things.

The performance analysis of anomaly detection is very subjective. The vast majority of approaches seen here attempt to rectify this issue by measuring the performance across commonly used metrics in machine learning. The works surveyed here utilize multiple performance metrics. All are reported in Table A1, Table A2 and Table A3. Each of the metrics used relies on four basic measures:

- True positives: the number of data points classified as anomalous that were truly anomalous;
- False positives: the number of data points classified as anomalous that were truly non-anomalous;
- True negatives: the number of data points classified as non-anomalous that were truly non-anomalous;
- False negatives: the number of data points classified as non-anomalous that were truly anomalous.

Here, we highlight the most-popular metrics, each of which relies on the above four basic measures:

- precision=TP/TP+FP;
- recall=TP/TP+FN;
- F1=2*((precision*recall)/(precision+recall)).

Precision is useful for determining how well what you detect is really there. The closer to 1 (100%), the better. Recall is useful for determining how well you capture detections and avoid misses. The closer to 1 (100%), the better. The F1-score is a combination metric that leverages these two metrics together and is a universal scorer for performance evaluation. ROC curves are a measure of how the true positive rates and false positive rates are compared. The TPR captures all actual positives in the system as a metric. The TPR is also known as recall. The FPR captures the number of actual negatives in the system as a metric. The FPR can be calculated as 1-recall.

Since this survey covers multiple applications and data-sets involving anomaly detection in sensor networks, a direct comparison of how well one approach compares to another is not always viable. However, one important aspect in any machine-learning-based approach to anomaly detection is generality to the application. Some methods, such as [15], were developed for specific applications, while other approaches, such as [14], were tested and trained on multiple data-sets. This cross-testing can help make the approach more generalizable to different applications, potentially at the expense of performance for a specific approach. This is a well-known trade-off between under-fitting and over-fitting an ML approach to a data-set or group of data-sets. Those approaches that utilize multiple data-sets in their research can be seen in Table A3. For those approaches that perform AD on the same data-sets, a comparison of the performance between approaches will be given.

In general, the best approaches seen in this survey rely on some combination of a neural network, with some attention mechanism to capture dependencies in time series data. Applications that utilize graph-based deep learning approaches are able to capture potential dependencies between IoT devices when compared to those that either ignore the natural graph-based layout of sensor networks or treat all data as coming from a single sensor. The breakdown of each approach, the methods used, and the applications the methods were used against will be seen in the following sections.

The rest of the paper is organized as follows: Section 2 focuses on related surveys in anomaly detection. Section 3 breaks down the surveyed works by application (the task they performed). Section 4 breaks down the surveyed works by the AD approaches used. Section 5 compares the performance of the AD approaches surveyed against one another if AD was performed on the same data-set, with an overview of the performance by application type. Section 6 outlines remaining problems and opportunities for AD in sensor networks and provides a synopsis of potential future work. Finally, Section 7 provides a recap and conclusion to this survey. An Appendix is available, which includes detailed tables for the models surveyed here, as well as the data-sets used by these models and a brief introduction to the structures and uses for various GNNs provided for context and as an illustrative reference.

## 2. Related Work

### 2.1. Related Surveys

Here, we highlight other surveys in the field of anomaly detection and IoT/sensor networks. We consolidated these related surveys into a quick-reference based on the problem focus of each survey in Table 1.

The work in [6] focuses on cloud computing in cloud computing environments. The paper highlights the length of time anomaly detection has been performed, with the earliest reference of anomaly detection (on facial recognition) being performed in 1990 [16,17]. This survey looked at research from arXiv, SpringerLink, and Web of Science. It focused on which AD methods were used, what purposes AD was used for, and how research has evolved over time. It breaks application areas into the following: intrusion detection, performance monitoring, failure detection, and root cause analysis. The vast majority of the data-sets from this study are related to intrusion detection.

The work in [1] focuses on anomaly detection using graph-based approaches with deep learning. It lists graph-based anomaly detection as “frontier research”. Technical challenges listed include a training objective for anomaly detection, anomaly interpretations, high training costs, and hyperparameter tuning (especially for unsupervised learning). This survey is broken down by anomaly detection type. This survey does not focus on the IoT, just graph-based data with deep learning.

The work in [3] refers to anomalies as “the discovery of outlying data/events in the data-set”. It also references determining anomalies by recognizing data that deviate from anormal patterns. It further defines anomalies as “an exceptional, abnormal, or unusual event”, and “rare, isolated, and/or surprising”. The paper labels two types of anomaly detection: proactive (for real-time data) and reactive (for post-processing data). It further splits anomalies into point, collective, and contextual types. This work focuses on non-time series data, so these definitions relate to the clustering of data. The authors split AD techniques into seven classes: classification, statistical distribution, graph, nearest neighbor, information theoretic, clustering, and spectral. For classification, they list the NN, SVM, Bayes network, and rule-based techniques. For nearest neighbor techniques, the KNN and relative density were mentioned. For clustering techniques, density-based clusters were mentioned (k-means and DBSCAN were popular in this set). For statistical techniques, parametric (such as linear regression) and non-parametric techniques (such as histogram-based techniques) were mentioned. The difference between the two is that parametric techniques rely on a statistical model to fit the data to and non-parametric techniques rely on real-world data to generate a model to fit test instances to. Information theoretic techniques focused on information gain and entropy-based techniques. Spectral techniques listed were primarily principal-component-analysis-based techniques. These techniques attempt to lower the dimensionality of the data to better separate nominal and anomalous data. For graph-based ADTs, outlier nodes (either highly connected or highly disconnected nodes) are focused on. Techniques in this group vary across the board from ML, such as SVM, to iForest and random forest techniques. This paper does not focus on any type of AD in particular and only contributes a small section to AD on graphs.

The work in [2] is a survey of anomaly detection in the IIoT and the GNNs used to perform the detection. It defines three types of anomalies: point, contextual, and collective. It gives examples of data-sets that exhibit that type of anomaly based on different problem domains. This work also has a good breakdown of GNN types, such as the GCN method for context-specific fault detection [18], a GCN for fault detection with collective anomalies [19], a combination of the GCN and LSTM into the RGCN to solve collective anomalies [20], a one-class GNN, which is a generalization of a one-class SVM [21], and AddGraph [22], which is an extended temporal GCN with attention used for capturing temporal patterns in dynamic graphs. In addition, this work explains the need for multiple GNNs to solve spatial and temporal anomalies. Finally, this work proposes an STGNN for collective anomalies in traffic data; a GCN is proposed for point anomalies in power Transformers; a GCN is proposed for collective anomalies in smart factory data.

The work in [23] focuses on anomaly detection for time series data. It focuses on multiple DL methods. Anomalies are listed as outliers, and it splits them into four categories: innovative, which affects data at and after some time *T* by some interference, additive, which only affects data (via interference) at time *T*, but not after, level shift, which permanently changes the structure of the data starting at time *T*, and temporary change, which affects the structure of the data at time *T*, but then drops off. This work surveys methods in the CNN, LSTM, AE, graph attention network (GNN), Transformer, global adversarial network (GAN), and DNN domains. A comparison of multiple DNNs on three datasets was also performed. It notes that the graph structure has great research significance in anomaly detection in time series. It also notes that base GNNs have a hard time adapting to dynamic graphs.

**Table 1:** Breakdown of application types for AD from related surveys.

Reference	Application	Focus
[6]	cloud computing	IDS
[5]	computer networks	IDS
[24]	malicious actor	space and information networks
[2]	IIoT	GNNs
[23]	attention-based AD	-
[4]	attention-based AD	-
[1]	non-IoT graph-based AD	deep learning
[3]	general AD	-

The work in [24] uses security and anomaly detection of space information networks (SINs), which are highly dynamic networks noticeably different in structure than traditional terrestrial networks as its focus. The anomalies focused on are mainly based on attacks on the network, although it lists other issues such as environmental and natural threats. The work surveys routing techniques in addition to anomaly detection techniques used in SINs. For AD, it is defined as finding exceptional patterns in a network that do not conform to the expected normal behavior. This work classifies an SIN as a dynamic network and, therefore, groups AD techniques for it in the same realm as social networks, citation networks, electric power grids, and global financial systems. It lists four types of anomaly: vertices, edge, subgraph, and event. Of these, it lists the subgraph and event types as unique to dynamic networks. This work proposes an anomaly detection scheme using a cybersecurity knowledge graph, with a GCN to encode the data. Decoding is performed in parallel for the structure and attributes using sigmoid and ReLU, respectively. These values are then aggregated into a D-value for the anomaly score. The survey points out that, while this looks promising, SINs are very complicated, and this is an active area of research. This work lists many future directions where SIN-based AD can go, including a uniform SIN security architecture and modeling, secure space–air–ground computing, blockchain-based applications, and lightweight crypto algorithms and protocols. This work also lists several SIN simulation platforms.

The work in [5] defines AD as “finding patterns in a data-set whose behavior is not normal or expected”. This work’s motivation is a robust IDS for computer networks. It focuses on data mining techniques, and defines four classes of technique for AD: association-rule learning, clustering, classification, and regression. The clustering algorithms mentioned are K-means, K-medoids, EM clustering, and outlier detection (distance-based (nearest neighbor) and density-based). The classification algorithms are the classification tree (ID3 and C4.5), fuzzy logic, naive Bayes, genetic algorithm, neural network (MLP), SVM, as well as hybrid approaches (cascading supervised techniques (NB + ID3, and DT + SVM) and supervised + unsupervised (KM + ID3, ANN + SVM)). This work has a good breakdown of non-DL AD techniques.

The work in [4] focuses on understanding AD using LSTM approaches. The motivation was the lack of studies with these approaches. It defines an anomaly as “a deviation from a rule, or an irregularity that is not part of the system behavior”. It uses this definition as a common ground across multiple AD research areas. It calls out network security, IoT, medicine, and manufacturing as AD research areas. It does not focus on a particular AD type, but rather, how LSTMs are used in AD. This paper characterizes anomalies as either focus points, some measured event, or some linearity/nonlinearity in the data and further lists that focus points can be on individual sensors, up to a whole system’s dynamics, and that the measurability of an anomaly can either be direct or indirect. The focus being on LSTMs, this work deals with time-dependent anomalies and specifies that anomalies are either long- or short-term and either stationary or non-stationary. This paper further elaborates on both collective and contextual anomalies mentioned in other literature. This paper highlights the benefits of LSTM models in modeling multivariate time series and time-variant systems. It also provides a good explanation of the LSTM cell. The work specifies how graph-based approaches using LSTMs improve the representation of contextual information.

### 2.2. Graph-Based Data Use

Here, we highlight some work that, while not on anomaly detection, highlights the usefulness of graph-based representations of data and IoT devices. The work in [25] highlighted a useful use-case of IoT-based data: water monitoring using low-cost multi-sensor IoT devices. The work in [26] expanded on the LMST algorithm [27] for determining the topology of an IoT network at the node level and finding the minimum power required to do so. The paper highlighted the usefulness of looking at an IoT (sensor) network as a graph. The work in [28] also highlighted the usefulness of viewing IoT data as a graph. It utilized Gumbel sampling on the IoT data. In [29], device fingerprinting using IAT graph plots was performed. Image classification using a CNN on those plots was then performed. The work in [30] looked at the “Heterogeneous Graph of Things” (HGoT). The authors treated nodes as heterogeneous. Then, the paper focused on node-level representation learning of the HGoT. They used an encoder–decoder to learn the relationships between heterogeneous nodes in the HGoT. The work in [31] used graph-based methods to detect patterns in IoT networks. The work in [32] involved the management of applications living on the IIoT. The work in [33] is a critique of the usefulness of GNNs (in particular, RouteNet) in the generalization of graph sizes. RouteNet is used to predict delay in a network topology. In [34], an IoT network was used to construct DNNs to perform ML. In [35], the focus was on the creation of a graph recovery model using a gated GCN, which sends information from available sensors to missing sensors in order to reconstruct their features. This work presented a useful breakdown of GCNs and GRUs. This work did not perform anomaly detection, but rather recovered data from missing sensors using GCNs. The work in [36] is a practical tutorial of GNNs with example applications. The work in [37] focused on utilizing differentiable network architecture search (DNAS) in order to find ML models suitable for small architectures such as the IoT.

## 3. Anomaly Detection by Application

The IoT and sensor networks are used for multiple different applications, most of which revolve around monitoring of some kind, whether that be monitoring on personal health devices, monitoring an entire city, monitoring the weather, monitoring an industrial system, etc. Anomaly detection in this context can fall into the following categories:

- Malicious actor AD;
- Sensor performance AD;
- Time series data AD;
- Other AD.

Some works cite more than one application type as a motivation. However, they are labeled by their primary motivation. A primary concern for most users of networks of devices, malicious actor detection, is an incredibly popular topic of research. Malicious actor AD can range from general-purpose intrusion detection systems (IDSs), to malware analysis, to specific malicious actors such as botnets. In addition, sensor network data can be heavily reliant on historical readings; for instance, a gradual increase in temperature may be normal, but a rapid increase may indicate an issue. This is colloquially referred to as time series data. Sensor performance AD is defined here as fault detection in a sensor network, as well as the performance in the detection of uncommon environmental changes. For instance, if a sensor network is trained to learn the normal operating characteristics of a system (such as nominal operating temperatures) and some environmental effect causes the system to fall outside of those operating ranges, the AD approach should be able to detect this anomaly successfully. Here, the difference between time series data AD and sensor performance AD relies explicitly on the need for attention mechanisms in the former case in order to find dependencies between data points.

Other problems for AD range from damage assessment in fuel pipelines, to distributed AD (on edge networks), to general-purpose anomaly detection. All works surveyed in this section have their application, primary focus, and secondary focus (if applicable) listed in Table 2.

The work [38] performed the detection of malicious attacks on a sensor network. In particular, the authors were looking at the detection of denial of service attacks on a network of IP cameras.

The motivation for [39] was intrusion detection in the IoT. As a result, the focus was on low-level sensor anomalies and application-level data (rather than network packet information). The authors built a framework due to the lack of available IoT data (the framework allows a user to generate a custom data-set), as well as the ability to test anomaly detection algorithms safely; most malware detection research relies on working with files and executables, which may contain active malware.

The work in [40] focused on DDoS attacks. Every IoT device was treated as an aggregate host device. As a result, this work did not look at the potential additional influence the different, individual IoT devices may have on DDoS-like anomalies (for instance, devices closer to the edge of the network, device type, etc.).

The work in [41] focused on SYN attacks on the IoT. All of the data were randomly generated from a virtual network composed of an unspecified number of nodes. Spatial position in the network was considered as well.

The work in [11] used graph-theoretic approaches to produce printable string information graphs (PSIGs) for IoT botnet detection. A PSIG is a trimmed function call graph that only relies on functions using string data. These PSIGs are made from IoT device executables. The inspiration for this work was using graph-based approaches for the detection of botnet attacks on IoT devices. The authors relied on static approaches (producing PSIGs from executables), rather than dynamic approaches (executing the malware IoT device in a sandbox) due to their view that, because IoT devices themselves are unsophisticated (limited power, storage, and computation), the botnets running on these devices are not sophisticated either.

The work in [42] used network-based malicious attacks on IoT devices as their inspiration. It used sample IP flows to obtain sparse data for anomaly detection. This technique explicitly calls out that it does not use the packet’s payload (i.e., the message sent from one IoT device to another). The goal of this work was to utilize a “transfer learning” technique by normalizing the losses of the DL network of each client network they train on using a training baseline, the goal being a network-agnostic anomaly detection approach.

The work in [14] focused on multi-variate time series anomaly detection. This work assumed anomalies will occur in contiguous windows and classified a whole window as anomalous if an anomaly is detected anywhere in the window. Their motivation was the protection of vital IoT networks such as smart power grids and water distribution networks.

For the work performed in [43], malware detection was the primary driver. The source code of different IoT architectures was converted into function call graphs (FCGs), so anomaly detection using deep learning methods could be performed.

In [12], the motivation was the protection of IoT and IIoT systems from attacks. The authors pointed out that, as more and more devices become Internet-connected and connect to one another, an additional risk for malicious attack is presented. In addition, as more of these devices connect to the Internet, they begin to produce larger and larger quantities of data. This exponential increase in data makes it impossible for a human to efficiently process it all. The authors utilized directed graphs, where the edges represent the dependency between sensors to show the context between IoT devices to assist in anomaly detection.

The work in [44] focused on the detection and prevention of attacks and intrusions on IoT devices. This work also mentions the lack of available data-sets for IoT devices. This study treated all IoT devices as a single large IoT device, rather than leveraging the graph-based nature of IoT networks.

For [10], the motivation was also attack detection in IoT networks. System errors were not listed as a type of anomaly in this paper. If data were missing from a row (represented by NaN values), each NaN was converted into a malicious value and that row was labeled an anomaly. This work also removed the timestamp column from the data-set used and, therefore, was not interested in time series data. In [45], the focus was on utilizing deep learning to develop an attack detection model for the IoT using network data. This work utilized the same data-set as [10], and the data-set was pre-processed to fill in missing data with meaningful information as well.

In [46], the authors pointed out that, as IoT device usage rises, malicious attacks on these devices will also rise. Their goal was to provide an efficient AD mechanism for IoT devices regardless of the ML model used. As such, they focused more on the selection of features from a data-set rather than the ML model itself. The data-set they utilized in their research was the Bot-IoT data-set.

The work in [47] worked with the IoT in water systems. Their motivation was the global water crisis low-income communities are facing. The goal was to utilize time series data from smart water systems to detect anomalies such as leaks, meter failures, illegal water use, warning situations, and peak water use.

The work in [48] looked at anomaly detection in a network of charging stations. Overall, there were 10 charging stations, 1 faulted and 9 normal, with 24hr logs over 20 days (plus a random bot sending OCPP-compliant messages). The logs contained requests and responses from a central server. Multiple different message types were present in the logs. The goal was to attempt to detect faults from these messages.

In [49], anomalies occurring in nuclear power plants was the focus. In addition, this work referenced [50], which is a 2009 anomaly detection survey, further showing the interest anomaly detection has received in the last 20 years. The data for this work were simulated using the SIMULATE-3K tool.

The work in [51] focused on fault detection in the structure of a network of sensors utilizing non-heterogeneous fault alarms. Bi-partite graphs were used to process the data. When multiple faults in a sensor network occurred at the same time, the resultant alarms that sounded (at the monitor stations) were not heterogeneous (meaning the sets of alarms that went off did not always designate the same set of faults). As a result, when multiple faults occur at the same time, the destination of the fault cannot always be found.

The motivation of [52] was real-time, efficient anomaly detection in edge networks for the IoT. This work pointed out the need for real-time AD in large sensor networks while avoiding large numbers of false alarms and large numbers of undetected anomalies. It also pointed out the pitfalls of using traditional NN approaches due to their inefficiency for detecting new anomalies in real-time.

In step with [52], the work in [53]focused on distributed anomaly detection, citing the incredible increase in IoT devices over the last few years and subsequent increase in load on cloud computing systems. The primary motivation in [53] was to handle anomalies in real-time, rather than wait for an analysis to occur at the cloud level. The Internet of Vehicles (IoV) (a subset of IoT) producing anomalous sensor data was used as a motivating example.

The work in [54] focused on the Industrial IoT (IIoT). The application focused on time series data produced by industrial devices. Specifically, the focus was on dynamic, normal (drift) operations in the IIoT. In situations such as these, what defines “normal” changes over time. As such, this work aimed to detect anomalies during “drifts” from stable (or “normal”) windows of time.

The motivation of [7] was industrial devices that rely on and produce multivariate time series data. Time series data are defined here as sensors producing data regularly over time. The multivariate component comes into play when multiple systems produce multiple data streams (i.e., from different sensors in the system). This work looked at multivariate time series data on a per-entity basis (many entities in the network and many sensors per entity). These entities produced large sets of data, which can have per-sensor anomalies, with some events (such as overloads) causing anomalies across all sensors in an entity. This work defines an anomaly as an unexpected incidence significantly deviating from the normal patterns formed by the majority of the data-set.

The work in [13] focused on multi-sensor time series data where the data do not always conform to a fixed input size. Their goal was to produce a model that enhances anomaly detection (compared to other techniques) in cases where sensors may not be available at particular time instances in some cases, but available in others.

The work in [55] focused on time series in the IIoT. Their motivation was the complexity and inter-connectedness of sensor data in IIoT systems. Their goal was to derive an efficient outlier detection framework that can handle time series data and detect anomalies with long-term, short-term, and weak time dependence. The data in this work was down-sampled for dimension reduction.

For [56], the motivation was to provide a robust anomaly detection capability for connected and automated vehicles (CAVs), namely to do so despite the lack of anomalous data present in the data. In addition, this work defines the following anomaly types: instant, constant, gradual drift, and bias.

**Table 2:** Breakdown of application types for IoT-based AD.

Reference	Application	Primary Focus	Secondary Focus
[38]	malicious actor	IDS	-
[39]	malicious actor	IDS	-
[40]	malicious actor	DDoS	-
[41]	malicious actor	SYN attack	-
[11]	malicious actor	botnet	-
[42]	malicious actor	-	-
[14]	malicious actor	-	time series data
[43]	malicious actor	malware detection	-
[46]	malicious actor	feature selection	-
[12]	malicious actor	-	-
[44]	malicious actor	IDS	-
[10]	malicious actor	IDS	-
[45]	malicious actor	IDS	-
[47]	sensor performance	water systems	time series data
[48]	sensor performance	charging system	-
[49]	sensor performance	nuclear power plant	-
[51]	sensor performance	edge connection fault detection	-
[15]	sensor performance	emergency detection	-
[54]	time series data	IIoT sensor drift	-
[7]	time series data	-	-
[13]	time series data	multivariate time series data	-
[55]	time series data	-	-
[57]	general AD	time series data	multi-class detection
[56]	general AD	automated vehicles	-
[58]	general AD	time series data	energy efficiency
[52]	distributed AD	-	-
[53]	distributed AD	time series data	-

The work in [15] utilized the IoT in the detection of emergencies in coal mines. It utilized environmental sensors connected in a network, including wind speed, Ch4, and CO sensors. It attempted to classify anomalous states in the network within the mine. The paper listed four “situations”, three of which are dangerous, thereby making this a multi-class classification problem.

The motivation for [57] was understanding anomaly detection in IoT networks. Their main focus was on multi-class anomaly detection utilizing time series data in the IoT. They pointed out that, due to the limited availability of labeled data-sets and the disproportionate occurrences of anomalous data in IoT devices versus nominal data, fully supervised approaches to general AD are not as successful as unsupervised approaches. On the other hand, the benefits of supervised approaches are missed when using purely unsupervised approaches. As a result, they utilized a hybrid semi-supervised approach to perform AD on a network of IoT sensors.

The motivation for [58] was trustworthiness, energy efficiency, and explainability for anomaly detection in the IIoT. They pointed out that there has been some progress toward explaining how AD is performed, but little to no work on making AD in ever-growing IIoT networks more energy efficient. This work relied on the trade-off between energy efficiency and accuracy. As such, applications that rely on more energy-efficient environments and that can handle less accurate results can utilize this approach by leaning more toward the energy efficiency side of this balance. The opposite is also possible. For the data-sets used, the authors relied on deductive imputation (using the mean of the feature column) to fill in missing values, and categorical data were converted from text to numerical data using LabelEncoder from SKLearn. SKLearn was also used to standardize the data. This was done so the results of the training were more generalizable.

## 4. Anomaly Detection by Approach

In addition to applications, anomaly detection can be performed with different ML (and non-ML) approaches, or combinations of approaches. Here, we break down our survey by approach used.

In [38], the authors relied on a symptomatic approach to anomaly detection. Using the logic that, when an attack is occurring on an IoT device, the device’s power consumption will rise, the approach used relies on recording the amperage of each IoT device (IP cameras in this work) during nominal use and during an attack. The authors ran their IoT network with normal operations, then began denial of service attacks on the nodes in the network, recording the amperage used by the nodes being attacked.

The work in [39] used the K-means algorithm for anomaly detection as a test case for a custom-designed framework for testing anomaly detection algorithms. In their test case with K-means, they built a 24 h simulated data-set to mimic a smart home network. The metrics included true and false negative/positive counts and the true positive rate (TPR). Cluster sizes in the K-means algorithm were varied, as was the cluster initialization criteria.

To perform the work in [40], an autoencoder was utilized. Since the data were viewed as a single sensor, rather than a network of sensors, the data were fed into the autoencoder to learn its signature, then decoded. Once training was complete, any new data fed into the autoencoder that looked like deviations from the trained model were considered as an anomaly.

The work in [41] looked at AD using two different types of networks, an LSTM and a Gelenbe network (also known as a random neural network or RANN). The LSTM model used was standard. The random neuron model used deviated from a standard perceptron in that, rather than a simple data input, the random neuron takes both an excitation signal and a inhibition signal (which are mapped to the input data). Both models were used as regressors to detect the normal state of the network. Then, as the test data, a dataset containing anomalous data was fed into the model, and the regressor’s prediction model would be off from nominal, resulting in the detection of an anomaly. In [41], node-level anomaly detection was performed. This was performed by utilizing the packet information about the IoT network. The goal was to detect SYN attacks, so this work utilized regression to predict the number of half-open TCP ports on the IoT device. If the number predicted deviated by some threshold, an anomaly was reported.

The work in [11] used multiple ML-based approaches such as Gaussian regression, SVM, decision trees, random forest approaches, and an MLP. Their approach relied mostly on the generation of PSIGs, rather than a new or novel classification model. The authors pulled 12 attributes from the ELF files of IoT devices, some at the graph level and some at the sub-graph level. At this point, this 12-dimensional information was passed into the listed classifiers to perform anomaly detection. The work in [11] examined data at the graph and sub-graph level of the ELF files of IoT devices themselves, not the IoT network. Information on how these devices were connected and may or may not be sharing information with one another was not considered. As such, their anomaly detection approach, while performing sensor-level classification, did so without consideration of the graph structure of the network, rather this was performed using the graph structure of the IoT device’s executable.

An autoencoder was chosen as the AD network in [42]. The justification for using an autoencoder here was due to the large reconstruction loss when seeing anomalous data when trained on strictly non-anomalous data. This justification was similar to other unsupervised techniques seen in this survey. This work claimed robustness against adversarial ML attacks due to how the DNN was implemented and the sparsity of the data collection. Normalization in the autoencoders was based on tasking (anomaly detection versus classification). For the former, min–max scaling was used, and for the later, norm scaling was used. This normalization of autoencoder loss was used to aid transfer learning across client networks.

The work in [14] used a connection learning policy based on the Gumbel softmax sampling trick (https://towardsdatascience.com/what-is-gumbel-softmax-7f6d9cdcb90e (accessed on 24 January 2023)) to overcome the quadratic complexity challenge and limits of this work. To perform their AD, they used a Transformer for its usefulness in capturing long-distance contextual information. Like other unsupervised approaches in this survey, their training was performed only on non-anomalous data, with prediction on data with anomalies.

The work in [43] used GraphSAGE and an MLP to perform malware detection on the IoT. The node features along with the FCGs generated from the IoT device architectures were fed into a GNN (GraphSAGE) to obtain the graph embeddings. This was then fed into an MLP for binary classification. The authors used multiple layers of GraphSAGE, summing the graph embeddings from each layer and using this as the input to the classification layer. Additional tests also included a GCN and hierarchical graph pooling with structure learning (HGP-SL) in place of GraphSAGE. For [43], function call graphs were used to represent source code on different IoT architectures. FCGs were extracted, and natural language processing was performed on blocks of opcode to obtain the embeddings, which were then used as the node features. Node-level classification using GNNs was then performed with the node features and FCGs.

In [12], unsupervised AD using a graph deviation network (GDN) was performed, with training only on non-anomalous data. The goal of the GDN was to detect patterns in the relationships between nodes on the network and report when there was a deviation from these relationship patterns. One-class classification on each tick of the time series data was performed. This work used embedding vectors based on the data-sets tested, whose weights were randomly set, then learned to represent unique features of a sensor. The attention coefficients were normalized using softmax. The anomalousness scores were normalized to avoid bias. The graph attention feature extractor used ReLU. The focus in [12] was anomaly detection on multivariate time series data. A sensor embedding containing the time series data and the number of sensors in the network was provided to two models: a relationship detection model and an attention-based forecasting model. Once the relationship model had run, that output was also fed into the forecasting model. The result was a graph deviation score, which was the determination whether any particular moment in time was anomalous or not. As a result, this work focused on whole system anomaly detection rather than anomaly detection on the sensor (node) level.

To perform AD in [44], both a standard MLP and logistic regression were used. To handle time series data, multiple time windows were added as parameters in the data-set. As all the data were treated as coming from one sensor rather than a network of sensors, potential information was lost due to ignoring the graph structure of the network.

The authors in [10] performed a comparison between multiple ML models and provided good explanations of some ML types and potential data-sets. Only one DL model was used (a standard MLP). Comparisons were against logistic regression, random forest, support vector machines, and decision trees. Label encoding was used to convert data into feature vectors. This kept the dimensionality of the data-set the same (rather than increasing the dimensionality, like other embedding vector functions often do). Like [10], the work in [45]also utilized a standard MLP for AD.

The authors in [46], rather than focusing on the ML model for anomaly detection, instead focused on data-set feature extraction. The motivation for this was to mitigate the cases where inaccurate features may cause misclassification of anomalies and to speed up the performance of AD models. To perform more accurate feature extraction, the authors expanded upon their work in [59] and utilized a combination of correlation attribute evaluation (CAE), and the AUC metric to select features from the data-set, they verified the importance of those features using the integration of TOPSIS and the Shannon entropy metric. This algorithm is referred to as CorrAUC. To perform AD, the features chosen by CorrAUC were fed into multiple different ML models and evaluated. Those models included the C4.5 decision tree, naive Bayes, random forest, and an SVM, with the C4.5 decision tree model performing best.

Rather than using NNs for anomaly detection, the work in [47] focused on the ARIMA and HOT-SAX algorithms. ARIMA used association rule learning for classification, and HOT-SAX used random forest. Both classification (anomaly and non-anomaly), as well as discrimination (anomaly type) were performed. The models without discrimination had high rates of false positives. The association rule discriminator was inconclusive due to the lack of available data. The data used were a custom data-set based on IIoT water meters and time series data, but they were treated independently (graph-based approaches were not used). The metrics used included accuracy, sensitivity specificity, and AUC-ROC.

In [48], a standard MLP was used for anomaly detection on the messages from the charging network. This was one of the few papers surveyed here that focused on fault-based AD, rather than malicious actor detection.

To perform AD, the data in [49] were pre-processed by applying the discreet wavelet transform (DWT) to the signals in the data, before feeding the output into a CNN. The DWT was chosen for its ability to capture both frequency and location information. This introduced an optimization problem into the mix, since a DWT relies on a “mother wavelet” in order to transform the data. The choice of this wavelet determines how the data are transformed. Detrending techniques [60] were also used on the signals prior to running through the DWT. There were in total 56 sensors used in the experiment.

To perform AD in [51], a custom Hopfield neural network (HNN) was used. An HNN has its neurons run in parallel (synchronously), as opposed to by layer (asynchronously), which helps reduce the computational complexity of fault detection as the number of faults and alarms in the problem increases. Because non-heterogeneous fault detection mechanisms were present, the work in [51] focused on multiple-fault detection in the network structures themselves, rather than the specific IoT devices (i.e., edge connection fault detection). As a result, whole graph detection was the focus in this work. When multiple faults occur at the same time, the destination of the fault cannot always be found, but [51] was made to detect that a fault has occurred. This was performed by generating a bi-partite graph between faults and detections, pre-processing the graph to remove obvious non-faults, then running the resultant graph through the custom HNN.

In [52], a cloud-based deep learning approach was used to generate graphs of both normal and anomalous patterns, and edge computing was performed for feature vector comparisons against both anomalous and normal patterns. This was performed by utilizing a threshold technique first with anomalous patterns, then with normal patterns. If neither passed a threshold, the resultant new data point went into the cloud for additional training. If normal and anomalous patterns were detected, aside from reporting the normal/anomalous pattern, the resultant patterns were stored in the cache on the edge node to be used as an additional comparison for future data inputs until the cloud platform can aggregate those patterns into new graphs. Anomaly detection was performed both on the edge nodes and the cloud using a heuristic that compared the feature vector of the input data to lists of known anomalous and non-anomalous data, as well as graphs of normal and anomalous data. These graphs seemed to contain nodes comprised of feature vectors. In [52], convolution was performed on sets of feature data, which seemed to consist of n-dimensional time series data, producing a 2D input. Then, linear classification was performed on the output of the convolver. Exactly how graphs were used was not explained in detail. In addition, how feature vectors were generated was not specified.

In [53], a distributed approach was utilized, where the analysis using a CNN-inspired rule-based classifier was used to update an anomaly rules database (ARB). This ARB was used by the edge nodes to detect anomalies in univariate and multivariate time series data. Training was performed using multiple real-world data-sets, as well as a synthetically generated data-set.

In [54], a recurrent neural network (RNN) was utilized, and the network was trained incrementally and was capable of detecting (using prediction error) sudden, incremental, gradual, periodic, and aperiodic drifts. This work used local window normalization (which is useful for non-stationary time series). In [54], because an RNN was used, the approach to AD was regression (prediction). As such, the RNN was used ot predict the next few time steps of time series data passed into the network. If the prediction was off, the data were anomalous. Anomalous data were data seen as independent of drift, so incremental training was only performed during drift, which can be seen as a gradual change in the stat of data over time, rather than a localized change.

In [7], an unsupervised AD model was used due to the anomaly diversity and lack of labels for training. This paper also took a look at anomaly interpretation, by looking at a given entity’s univariate time series data and choosing the data with the lowest reconstruction probabilities. To perform AD, this work utilized a custom DNN, OmniAnomaly, which is a stochastic RNN (GRU + VAE). The work in [7] also relied on an unsupervised regression to perform AD. They analyzed reconstruction probabilities in both the multivariate and univariate cases for each entity in the network to determine what was an anomaly and what was not. As such, anomalies were decided on a per-entity bases, rather than on the network as a whole.

AD in [13] was performed using a “Conditioning” GNN, fed into a GRU “core”. The time series data were pre-processed to obtain a sensor availability embedding, then run through the conditioning GNN. The result of that process produced a sensor availability vector, which was fed into the GRU as additional input along with the multivariate time series data (which were mean-imputed to fill in gaps where there was missing sensor data). In [13], the goal was anomaly detection on multivariate time series data. The authors allowed for classification (anomalous/non-anomalous) and regression to be used. The classification utilized a softmax output layer to the GRU, while a sigmoid function was used for regression. Since a GRU is made to handle data of a fixed input size, the sensor network was summed into a single IoT node. As such, the work in [13] provided anomaly detection based on a window of time, rather than a particular sensor (node).

An LSTM was used in [55] to both handle time series data and deal with vanishing and exploding gradients, which can arise with longer time windows in standard RNNs. Specifically, a stacked LSTM with Gaussian naive Bayes processing was used. This paper went into detail on how LSTM networks are constructed and work and can be a good reference material for models using LSTMs. Because time series data were the focus in [55] and an LSTM was utilized, each row in the data-set was one moment in a time window used in the LSTM processing. The anomaly detection model ran the data-set through the LSTM, ran the results through a Gaussian distribution (using maximum likelihood estimation), then, along with the LSTM results, passed the results of the Gaussian distribution through a naive Bayes model and reported on the presence of an anomaly.

The work in [56] focused on an ensemble approach for anomaly detection. This was performed by utilizing a CNN and LSTM for regression on the sensor data, then using an ensemble approached called WAVED, enhanced using this prediction for classification. WAVED is a combination of random forest, AdaBoost, and an SVM. In [56], multi-class classification was performed. The anomalies of interest were instant, constant, gradual drift, and bias. Because this work focused on a sensor network in a vehicle, the classification of anomalies was defined at the vehicle level, not the sensor level. The CNN extracted features from the sensor network, and the LSTM took those features to predict a signal. This was then fed into WAVED, which performed a majority voting rule from each of its ensemble classifiers, then this, along with the data prediction, determined the class of anomaly (or if the data were nominal).

To perform the anomaly detection in [15], an MLP was utilized. The authors called this a “BP” network for back-propagation, which is the standard function of an MLP. Only a single hidden layer was used in the MLP. In [15], the authors were interested in four classes for detection: no anomaly and Risk Grades 1, 2 and 3. To do this, the output layer of their MLP used two neurons. Their results (which were 1 or 0) indicated 1 of the 4 classes. The classification performed wassystem classification. Since the goal was safety detection in a coal mine, any anomaly detected anywhere in the system resulted in an alert to the whole system.

To perform AD in [57], the authors utilized a combination of an temporal convolutional network (TCN) and a variational autoencoder (VAE) on the DS2OS data-set. Their approach was called SS-VTCN, which was a semi-supervised approach taking into consideration partially labeled data during training, as well as an anomaly prediction score passed on to the VAE. In addition, this approach utilized rectification parameters when each side of the NN did not agree. Since the work performed in [57] was semi-supervised, it was a combination between sensor classification and regression, with the labeled training data being used to enhance the anomaly prediction. In order to avoid leakage from “future data”, the authors only allowed the NN to train on data that had gone through the trainer previously. In other words, the test data-set became “fresher” than the training data-set. The amount of labeled data was increased from 10% to 50% during the analysis of this approach. In addition, because this approach was semi-supervised, the anomaly detection performed by the VAE portion of SS-VTCN can be enhanced by utilizing the reconstruction probabilities of the labeled training data. Interestingly, because the labeled portion of SS-VTCN performed dimensionality reduction and because VAEs generally create lower-dimensional representations between the input and the reconstruction, the authors here modified their VAE to do the opposite: create a higher-dimensional intermediate layer to reconstruct lower-dimensional inputs.

In [58], feature extraction was performed using a standard autoencoder, with AD using a VAE and feature selection using DeepExplainer. A standard autoencoder’s encode and intermediate layers were used to extract features to perform AD on. This reduced the dimensionality of the data. From here, a VAE was used to perform AD, then DeepExplainer was used to perform feature selection for a re-run through the VAE. Regression-based anomaly prediction was performed in [58]. This work extracted the features using an autoencoder, predicted “first level” anomalies using a VAE, then utilized DeepExplainer (which is a modification of the Shapley additive explainer, which is a linear function of binary variables that helps determine which features used in an ML model are contributing toward the model output and Bayesian local explanations to capture the uncertainty from the explainer). The autoencoder was used for feature extraction (the encode and intermediate layer), as well as for the reduction of the computational complexity of the AD model. It was this reduction in complexity that contributed to the energy efficiency. To select the features of highest importance to re-run through the VAE to determine anomalous output, DeepExplainer works on concepts from game theory. In order to ensure good computational importance, rather than random sampling of perturbations, focused selection was used. DeepExplainer iterates from the two most-important features, to all extracted features, and re-runs the VAE, tracking the F1-score each time. The run with the highest F1-score was utilized. This was how this work handled the trustworthiness of the model.

## 5. Comparison of Performance

This survey focused on anomaly detection across multiple applications, methods, and approaches. However, the data-sets used in this survey were not all unique to each work. As such, there was some overlap in the AD methods surveyed and the data-sets they used. Here, we compare the performance of any AD approach that utilizes the same data-set.

Of all the works surveyed, eight papers used at least one data-set that was the same as at least one other paper. Table 3 breaks down the works surveyed and the similar data-sets between them.

SWaT is the Secure Water Treatment data-set and is a water treatment data-set collected across 11 days, which simulates cyber attacks on a water treatment system in the last 4 days of running. WaDI is a water distribution data-set, which is an extension of SWaT. WaDI contains 16 days of data with 2 days of simulated attacks.

The work in [12,14] utilized both of these data-sets and provided their results using the precision, recall, and F1-score. The work in [12] utilized a graph deviation network (GDN) composed of an embedding function, graph structure learning relying on directed graphs to capture the dependencies between sensors, an attention mechanism to forecast future values of the sensors, and a deviation scoring mechanism. The work in [14] also leveraged a directed graph structure learning policy and did so using the Gumbel softmax sampling strategy inspired by the policy learning network used in some reinforcement learning methods. In addition, the work in [14] leveraged techniques from graph convolutional networks (GCNs) and a customized multi-branch attention Transformer to capture time-related dependencies in the data.

The work in [14] reported multiple different versions of their metrics based on their best runs as defined by the recall (represented by *) and F1-score (represented by **). Metrics for each work for the SWaT data-set are reported in Table 4. Table 5 reports metrics for the WADI data-set.

In [14], more weight was given to the recall and F1-score. Their justification was that, in a real-world scenario, the tolerance of false alarms is preferable if better performance in detecting real anomalies is shown. If we use this as the context for comparison, the work in [14] significantly outperformed [12] in both recall and F1-score. This indicates that the GTA method of [14] provided a significant improvement over the state-of-the-art. The work in [12] was referenced in [14] for comparison purposes against these two data-sets and performed second-best when compared to the other comparison methods used by [14].

SMAP is a soil moisture data-set generated by satellite imagery, and MSL is a sensor data-set from the Mars Rover. Both of these data-sets are provided by NASA and contain labeled anomalies.

SMAP and MSL were used to evaluate the performance of [7,14]. In [7], a custom approach called OmniAnomaly was developed utilizing a vast array of optimization methods. It first starts with a gated recurrent unit (GRU), which is a simpler (but equivalently performing) attention network based on an LSTM. This was used to capture temporal dependencies in the data. Secondly, it utilizes a variational autoencoder (VAE) to map observations on input data to the resultant output. This VAE utilized a linear Gaussian state space model to model temporal dependencies in the latent space and planar normalizing flows to help capture complex distributions in the data. OmniAnomaly is split into an encoding (qnet) and decoding step (pnet) and uses reconstruction probability to determine if an anomaly is present.

Like the previous data-sets, the work in [7,14] utilized the precision, recall, and F1 as their reporting mechanisms. Unlike the previous data-sets, the work in [14] only reported their best scores based on the F1. Metrics for each work based on the SMAP data-set are reported in Table 6. Table 7 reports metrics on the MSL data-set.

For the SMAP data-set, taking into consideration the context that it is generally better to accept false positives than have false negatives, the work in [14] outperformed [7], although the margins compared to the previous data-sets and the comparison between [12,14] were much smaller. This was even further seen when looking at the MSL data-set. The work in [7,14] were within 2% of one another for the F1 and were identical for the recall. The work in [7] was also used as a comparison in [14] against the MSL and SMAP data-sets and either tied with [14] (for recall), was second-best (for precision), or came within 1% of second-best (for F1-score). This led us to believe that the GTA method of [14] was only marginally better than the existing OmniAnomaly method.

The determination of the complexity between these two methods is subjective, but each is generally more complex compared to more traditional deep learning methods such as MLP, LSTM, or CNN. Interestingly, the work in [14] compares itself to many less complex methods for SWaT and WaDI and more complex methods for SMAP and MSL. This led us to believe that SMAP and MSL may require more complex ML mechanisms to more accurately perform anomaly detection.

For [10,45,57], the only similar metric between them that was reported was the F1-score. For [45], an MLP was used to perform AD on the DS2OS data-set. For [10], multiple approaches, including logistic regression, decision tree, random forest, support vector machine, and an MLP were tested against DS2OS. For [57], multiple custom approaches were compared against a proposed semi-supervised variational temporal convolutional network (SS-VTCN), which is a TCN + VAE, on the DS2OS data-set. Since SS-VTCN is semi-supervised, different levels of data were labeled prior to training/testing. Here, we show the best performance of SS-VTCN against the MLP from [45] and all the ML approaches used in [10].

The DS2OS data-set has six different classes. All three works grouped those classes in different ways, and the only work that reported any information on the grouping was [57]. As a result, we treated the reported F1-scores as an average across all class groupings. MEtrics are reported in Table 8.

The work in [45] compared its proposed approach to other well-known approaches such as SVM, SGD, LDA, RF, LR, DT, and Gaussian NB, with its proposed approach performing comparatively similar to the other approaches. The work in [10] confirmed this, showing all of its ML approaches to be close in performance. For [57], a much more complex ML approach was used, and at best, it performed within a margin of error to more simpler approaches seen here on the same data-set.

We believe that this shows that data-sets like DS2OS do not need to rely on overly complex anomaly detection mechanisms for good performance.

In [52], an edge+cloud-based method was used called AdaGUM. At the edge level, anomalies were compared to a lookup table, as well as a graph-based representation of all known anomalies seen in the network. This was performed first for anomalous patterns, then for normal patterns. If no matches were found in either case, the data were sent to the cloud and a convolutional neural network (CNN) was used to perform the anomaly detection. The results of the CNN were used to update the nominal and anomaly records in the edge nodes.

In [44], a general MLP and logistic regression were used independently of one another and in parallel to perform anomaly detection.

**Figure 1:**
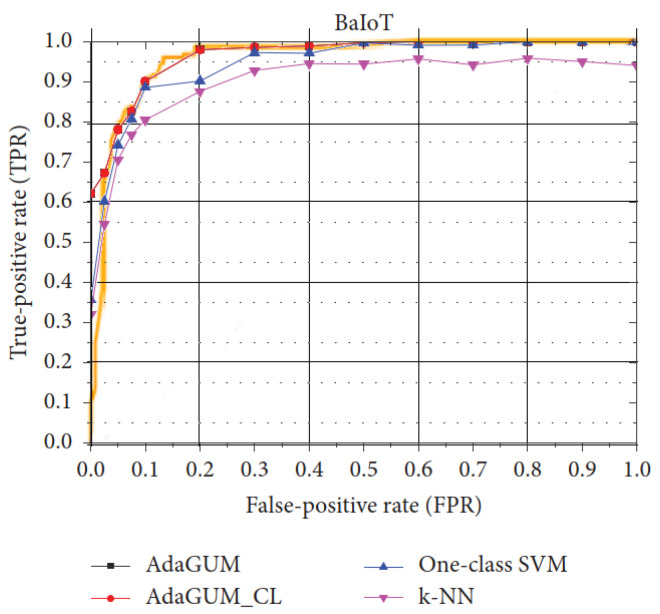
Comparison of ROC curves from [44,52].

For [44,52], the only comparison between the two of them was their ROC curves. As such, these figures were pulled from each paper and combined. Figure 1 shows the results. For [44], the proposed MLP performed worse than the logistic regression model used, so here, we show the logistic regression model of [44] against the AdaGUM model of [52], with related comparisons from [52] shown as well. As we can see, all models performed fairly well compared to one another, with [44,52] showing nearly identical performance. We believe this indicates that the BaIoT data-set is fairly easy to classify for machine learning models.

In addition to the direct comparisons we made earlier in this section, here, we show the general performance of each surveyed work based on their application type. This is not a direct comparison, as each work did not necessarily use the same data-set. We report (where applicable) the accuracy, precision, recall, and F1-score for each work surveyed in the tables based on the application. For any metric not available, a “-” is present. This is indicated where the data were averaged over multiple results. Papers without any of those metrics or papers that did not fall into a specific application category were omitted.

As we can see in Table 9, for the malicious attack application, very little (if any) performance is gained by producing highly complex models. In addition, how a model is connected can greatly effect the performance. This can be seen in the performance of the MLPs used in multiple works and the autoencoder in [40], where the combination of the two, as seen in [45], performed markedly worse. It should be noted here that a direct comparison between these works was not performed since the data processing and data-sets used differed.

The data for the time series application had performance ranges from the mid 80% to the high 90% ranges (based on the F1-score). This would indicate that there was some marginal improvement from more recent approaches such as SS-VTCN from [57] and GTA from [14]; however, the improvements seemed to be data-set dependent. Metrics can be seen in Table 10.

Each of the works in Table 11 performed anomaly detection on completely different networks of sensors. Interestingly, their accuracy was similar. Each model in Table 11 becomes more and more complex with little to no significant improvement. This may indicate that, like Table 9, more complex models do not necessarily produce better anomaly detection. Again, since each of these models was used on a different data-set with different data processing, a direct comparison is not wholly accurate, and these results should be used as a reference.

## 6. Research Directions and Opportunities

While this survey laid out the research being performed in anomaly detection, it also outlined gaps in the research. The availability of robust data-sets for sensor networks is hard to come by. Recent work on benchmarks such as Stanford’s Open Graph Benchmark (OGB) (https://github.com/snap-stanford/ogb, (accessed on 24 January 2023)) [61] are assisting with this deficiency. A future direction in the area of data-set generation can focus on the collection of IoT sensor data similar to what was done with Chicago’s *Array of Things* (https://arrayofthings.github.io/, (accessed on 24 January 2023)).

A practical concern for anomaly detection in the IoT is performing AD on the IoT devices themselves. The vast majority of ML approaches surveyed did not consider the execution restrictions of very small devices such as those used in the IoT. Of all the papers surveyed, only three were found to address this issue as a primary concern. Two of these approaches [52,53] were surveyed here. Another of these approaches [62], did not meet the criteria of this survey, but is worth mentioning here due to the unique nature of the research. In [62], a distributed anomaly detection network was created in order to perform node- and neighborhood-based AD in a network. Larger, edge AD systems performed the neighborhood anomaly detection, while smaller, node AD systems performed the node-level AD. This was done utilizing various sizes of MLPs at each layer. For a more robust AD framework for the IoT, the research being performed in mobile and edge machine learning, such as Voghoei et al’s “Deep learning at the edge” [63], can be useful.

A more theoretical concern in anomaly detection that needs more study is understanding what is going on between hidden layers in these neural networks related to the specific tasks of anomaly detection in the IoT. All the approaches surveyed here focused on some performance gain against a specific data-set compared to other AD approaches. Rather than focusing on iterative performance gain, understanding how a data point is being transformed and using that understanding to find better approaches to AD in IoT is an open area of research.

With regard to performance-driven AD in the IoT reviewed in this survey, the majority of ML used for anomaly detection relies on techniques that are not graph-based. This is due in part to the relative newness of graph-based techniques and also provides an opportunity for those wanting to perform research in this area. In particular, how graph-based anomaly detection techniques work on independent sensors is an open problem. Sensors in a network such as this may interact with one another, but are not explicitly required to. How they are connected (spatially, physically, through communications, etc.) is an area of active research.

Furthermore, most research being performed is on attack-based anomaly detection. While attack prevention in networks is extremely important, it is only part of anomaly detection. This provides an opportunity for more research to be performed in the areas of general AD for detecting multiple types of anomalies across the space or on performance-based AD, where a network’s anomalies are based on faulty equipment or abnormal environmental conditions, rather than malicious attack.

Furthermore, most network-based machine learning leverages the TCP-IP nature of Internet-connected devices. This usually means information being acted upon takes the form of packet data. This strict adherence to a TCP-IP structure may bias the data-sets in ways not currently studied. An open question is how/if the structure of the data being collected in a sensor network have an effect on the performance of a DL network, and both additional study using raw sensor output and the study of how strictly formatted sensor network data biases the results of DL networks (whether IoT data, network data, or not) are potential future avenues of study in the area of anomaly detection.

Lastly, work focusing on distributed AD in IoT similar to [52,53] and edge computing AD similar to [64] in the IoT is scarce. With the proliferation of the IoT at home, on our person, in the workplace, and in the environment, research on the efficiency/performance of distributed AD and edge-computing AD are in a prime position for expanding the state-of-the-art and improving AD overall in the area of the IoT.

## 7. Conclusions

For a complete breakdown of all the hyperparameters, each deep learning approach used, and the data-sets, please see Table A1, Table A2 and Table A3.

Anomaly detection in the IoT over the past few years has been leveraging multiple different approaches. Surveyed here were 10 non-DL AD approaches, 10 approaches that utilized an MLP to perform AD, 6 approaches that used graph-based techniques, 4 approaches that utilized attention-based techniques such as LSTM and GRU, 7 approaches that used encoder approaches such as the autoencoder or VAE, 4 approaches that utilized a CNN, and 6 approaches that used custom neural networks. This indicates the usefulness of deep learning and other machine learning techniques across multiple applications for anomaly detection in the IoT, despite the approach used. Many approaches utilized either an MLP or GNN as the base for the network, then utilized some other component (such as an encoder or RNN) for additional performance.

The approaches surveyed here were also varied: 9 approaches were concerned with time series data (attention-based AD), 6 on sensor performance, 2 on distributed AD, 13 on malicious attacks in the IoT, and 3 on general AD. Notice that the vast majority of sensor network and IoT AD focuses on malicious actor detection as a primary application/motivation.

Multiple data-sets across different focuses were cited by the papers in this survey. Table A4 lists all readily available data-sets referenced by works cited in this survey. They are grouped by problem focus. Good resources for additional ML/DL and AD data-sets can be found at Papers With Code (https://paperswithcode.com, (accessed on 24 January 2023)), Kaggle (https://www.kaggle.com/datasets, (accessed on 24 January 2023)), and UCS Datasets (https://archive.ics.uci.edu/ml/datasets.php, (accessed on 24 January 2023)).

To conclude, this survey covered anomaly detection (AD) techniques for sensor networks, with a primary focus on the IoT and graph-based data representation. Most works surveyed fell between 2017 and 2022 due to the large increase in both ML techniques during that time, as well as a large interest in AD using deep learning. As seen here, much of the work utilized more “standard” ML approaches such as MLPs, RNNs, or encoders or non-deep learning approaches such as logistic regression, SVM, or tree-based techniques, while newer approaches have been attempting a fusion of these approaches and incorporating graph-based learning techniques.

## Figures and Tables

**Table 3:** Data-sets and the surveyed AD works that used them. An ’X’ indicates if a work utilized a data-set.

Dataset/Cited Paper	[12]	[14]	[7]	[45]	[10]	[57]	[52]	[44]
SWaT	X	X	-	-	-	-	-	-
WaDI	X	X	-	-	-	-	-	-
MSL	-	X	X	-	-	-	-	-
SMAP	-	X	X	-	-	-	-	-
DS2OS	-	-	-	X	X	X	-	-
BaIoT	-	-	-	-	-	-	X	X

**Table 4:** Comparison of cited works using the SWaT data-set. * Indicates reported results based on recall. ** Indicates reported results based on F1 score.

Reference	Precision	Recall	F1
[12]	99.35	68.12	0.81
[14] *	74.91	96.41	0.84
[14] **	94.83	88.1	0.91

**Table 5:** Comparison of cited works using the WaDI data-set. * Indicates reported results based on recall. ** Indicates reported results based on F1 score.

Reference	Precision	Recall	F1
[12]	97.50	40.19	0.57
[14] *	74.56	90.50	0.82
[14] **	83.91	83.61	0.84

**Table 6:** Comparison of cited works using the SMAP data-set.

Reference	Precision	Recall	F1
[14]	89.11	91.76	0.9041
[7]	74.16	97.76	0.8434

**Table 7:** Comparison of cited works using the MSL data-set.

Reference	Precision	Recall	F1
[14]	91.04	91.17	0.9111
[7]	88.67	91.17	0.8989

**Table 8:** Comparison of cited works using the DS2OS data-set.

Reference	Approach	F1
[45]	MLP	0.98
[10]	LR	0.98
[10]	SVM	0.98
[10]	DT	0.99
[10]	RF	0.99
[10]	MLP	0.99
[57]	SS-TCVN	0.9616

**Table 9:** Performance of surveyed works for malicious attack application. An * indicates averaged data.

Reference	Approach	Accuracy	Precision	Recall	F1
[40]	autoencoder	-	0.994	0.941	0.967
[10]	MLP	0.994	0.99	0.99	0.99
[41]	LSTM	0.627	-	-	-
[41]	Gelenbe Network	0.807	-	-	-
[11]	Gaussian (regression)	0.91	-	-	-
[11]	Gaussian (classification)	0.9418	-	-	-
[11]	Decision Tree	0.9418	-	-	-
[11]	Random Forest	0.9484	-	-	-
[11]	SVM	0.9314	-	-	-
[11]	MLP	0.939	-	-	-
[44]	MLP	0.964	0.939	0.951	0.9913
[44]	Logistic Regression	0.9998	0.999	0.9996	0.9992
[12]	GDN (SWaT)	-	0.9935	0.6812	0.81
[12]	GDN (WaDI)	-	0.9750	0.4019	0.57
[42]	autoencoder + MLP	-	0.58	0.15	0.24
[43]	GraphSAGE + MLP	0.9961	-	0.9971	-
[45]	MLP	0.9828	0.97	0.98	0.98
[46] *	C4.5	0.9998	0.9711	0.9428	0.9567

**Table 10:** Performance of surveyed works for time series data application. An * indicates averaged data.

Reference	Approach	Accuracy	Precision	Recall	F1
[7] (SMAP)	OmniAnomaly	-	0.7416	0.9776	0.8434
[7] (MSL)	OmniAnomaly	-	0.8867	0.9117	0.8989
[7] (SMD)	OmniAnomaly	-	0.8334	0.9449	0.8857
[47]	HOT-SAX	0.76087	0.57143	0.85714	0.68571
[55] *	LSTM-GNB	0.9637	0.9547	0.956	0.9523
[14] (SWaT *)	GTA	-	0.8487	0.9226	0.875
[14] (WaDI *)	GTA	-	0.7924	0.9156	0.83
[14] (SMAP)	GTA	-	0.8911	0.9176	0.9041
[14] (MSL)	GTA	-	0.9104	0.9117	0.9111
[57]	SS-VTCN	-	-	-	0.9616

**Table 11:** Performance of surveyed works for sensor performance application. Values were averaged due to multiple results being reported.

Reference	Approach	Accuracy	Precision	Recall	F1
[48]	MLP	0.9383	-	-	-
[49]	CNN + MLP	0.9687	-	-	-
[56]	MSALSTM-CNN	0.9377	0.9207	0.9846	0.9512

**Figure A1:**
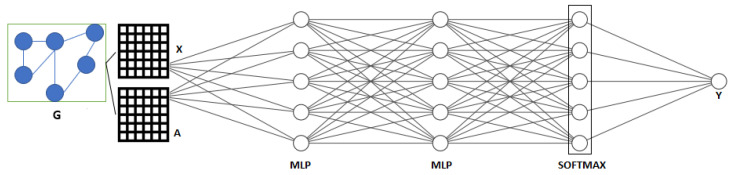
An example of a basic GNN.

**Figure A2:**
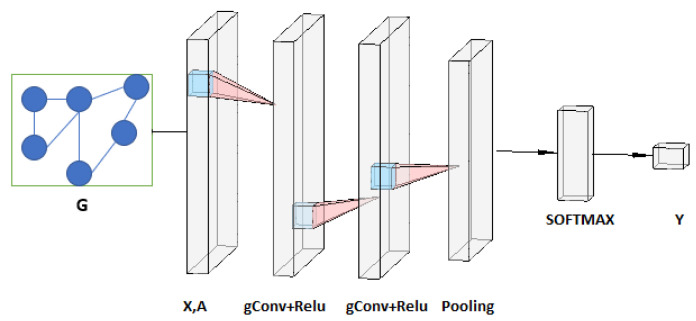
An example of a convolutional graph neural network.

**Figure A3:**
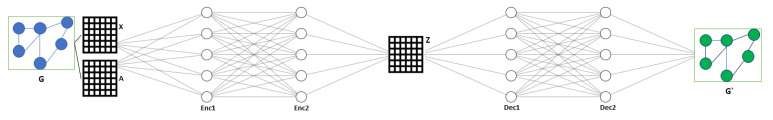
An example of a graph autoencoder (GAE).

## Data Availability

Not applicable.

## Appendix A. Dataset and Breakdown of Approaches

Table A1, Table A2 and Table A3 are a breakdown of the anomaly detection approaches, their hyperparameters, and their metrics. If a hyperparameter or NN component was not specified, the respective cell is left blank. Table A4 lists all the data-sets used in the works surveyed here.

**Table A1:** Breakdown of ML types surveyed.

Reference	Source	Year	NN Type(s)	Layers
[38]	ACM	2017	NOT NN: amperage recording	
[39]	ACM	2020	NOT NN: K-means	
[12]	AAAI	2021	GDN	30 (max)
[48]	ACM	2017	MLP	3
[54]	ACM	2018	RNN-AD	4 (max)
[40]	ACM	2019	autoencoder	5
[49]	ACM	2019	CNN + FC	6
[7]	ACM	2019	OmniAnomaly (GRU + VAE)	4
[11]	ACM	2021	Gaussian regression, SVM, decision tree, random forest, MLP	5
[42]	ACM	2021	normalized autoencoder (local), MLP (global)	9 (max)
[41]	ACM	2020	LSTM,Gelenbe Network	4,3
[57]	ACM	2022	SS-VTCN (TCN + VAE)	8
[58]	ACM	2022	AE+VAE	5 (VAE: 2enc, 2dec +1)
[13]	arXiv	2020	GNN with GRU	5
[15]	IEEE Xplore	2013	MLP	3
[51]	IEEE Xplore	2019	Custom Hopfield NN	5
[28]	IEEE Xplore	2019	MLP encoder/decoder with Gumbel sampling	
[47]	IEEE Xplore	2019	NOT NN, NOT graph-based	
[56]	IEEE Xplore	2020	MSALSTM-CNN	5
[55]	IEEE Xplore	2020	Stacked LSTM with Gaussian naive Bayes processing	4
[65]	IEEE Xplore	2020		
[3]	IEEE Xplore	2020		
[44]	IEEE Xplore	2021	NN and logistic regression (ensemble)	5
[2]	IEEE Xplore	2021	STGNN, GCN	3+N (max)
[14]	IEEE Xplore	2021	GTA	l5
[43]	IEEE Xplore	2021	GraphSAGE, MLP	K+1
[23]	*Journal of Physics*	2021		
[24]	*Nature*	2021	GCN (encoder)	
[10]	Science Direct	2019	MLP	
[45]	Wiley	2021	MLP	6 (max)
[52]	Wiley	2021	CNN, MLP	

**Table A2:** Breakdown of hyperparameters in surveyed works.

Reference		Largest Layer	Activation Function	Classifier/Regressor	Training Epochs
[39]					24 h of data
[12]		128	Leaky ReLU		50
[48]		5	threshold		
[54]		40	sigmoid and tanh		
[40]		14	ReLU		100
[49]		256			
[7]			sigmoid, ReLU, softplus	GRU	
[11]		12	SeLU		
[42]		60	ReLU	LSTM, sigmoid, softmax	
[41]		50			
[57]		8		softmax/regression	8
[58]		50-100	leaky ReLU (AE), ReLU+Lin (VAE enc), ReLU+Sig (VAE dec)	Regression	100
[13]		128	leaky ReLU, max, ReLU	max, softmax, sigmoid	150 (trn), 50 (f-t)
[15]		15	logarithmic		7
[51]		10	tanh, sigmoid		5-80
[28]					500
[56]		64	ReLU, tanh		200
[55]		varies	sigmoid, tanh		
[44]		40	ReLU	sigmoid	100
[2]			ReLU	softmax	
[14]		128			50 (5x mean)
[43]		varies	avg pooling, ReLU, max pooling	Softmax	
[23]					
[24]			sigmoid, ReLU		
[10]			sigmoid, ReLU, Leaky ReLU, tanh	softmax	
[45]		35	ReLU	softmax	300
[52]			ReLU	linear 1-class	
[12]	0.001			MSE	Adam
[48]	0.005				
[54]	0.0005		0.02 dropout	min avg pred err	
[40]	keras default	32		MSE	Adam
[49]	0.0001		0.35, 0.3 dropout	cross-entropy	Adam
[7]			Kullback–Leibler loss	negative reconstruction err + reg	
[42]	grid search opt		dropout (grid search opt)	MAE, cross-entropy (binary, categorical)	Adam
[41]				MSE	Adam
[57]	0.005			modified log (TCN), reconstruction loss with KLD (VAE)	Adam
[58]		mini batch		reconstruction loss using MSE and KLD	Adam
[13]	0.0005, 0.0001	64, 32(trn), 32(f-t)	0.2 dropout	cross-entropy, squared error	SGD, Adam
[15]	0.1			MSE	MATLAB trainlm
[28]				KL-divergence+ MSE	
[56]	0.025	200	L2 w/0.2 dropout		Adam
[55]	varies	varies	dropout, varies	MSE	GD
[44]				log log	GD
[2]					batch GD
[14]	0.0001	window size = 6	0.05 dropout	MSE	Adam
[43]	0.001			cross-entropy	Adam
[24]				D-value (anomaly score)	
[10]				cross-entropy	coordinate descent, GD
[45]			0.4 dropout	negative log likelihood	Adam
[52]			batch norm w/rand dropout		

**Table A3:** Breakdown of data-sets and metrics used by surveyed works.

Reference	Data-Set(s)	Metrics
[38]	IP camera amperage	amperage
[39]	24 h of data from custom smart home	TN, FN, TP, FP counts and associated TPR
[12]	SWaT, WaDI	precision, recall, F1
[48]	charging station logs	accuracy, F1
[54]	NAB, and yahoo variants, real-world server job	AUC
[40]	benign IoT traffic, generated DDoS	precision, recall, F1
[49]	nuclear power plant data (from SIMULATE-3K)	weighted accuracy
[7]	SMAP, MSL, SMD, custom (server dataset)	precision, recall, F1
[11]	IoTPOT, VirusShare, custom GitHub (benign), IoT ELF files (benign)	accuracy, FNR, FPR
[42]	proprietary real-world set, UNB2018, UNB2019, UNB2012	precision, recall, F1
[41]	custom SYN attack for virtual IoT network	accuracy, FPR
[57]	DS2OS	F1, class-based average F1
[58]	SECOM, wafer manufacturing, APS failure	precision, recall, F1, FPR, wall clock time (for energy consumption)
[13]	DSADS, HAR, Turbofan	classification error, RMSE
[15]	coal mine data (private)	
[51]	custom	fault location rate
[28]	air conditioning (private)	
[47]	custom water meter	accuracy, AUC, sensitivity, specificity
[56]	Wyk et al. 2020 reference	accuracy, precision, F1, sensitivity
[55]	power, loop sensor, land sensor	accuracy, precision, recall, F1, ROC, AUC
[3]	many, including KDDCup99	
[44]	BaIoT	accuracy, recall, F1, FPR, TPR, specificity
[2]	multiple	
[14]	SWAT, WaDI, SMAP, MSL	recall, precision, F1
[43]	IoT malware/benignware	accuracy, recall, ROC, AUC, classification time per sample
[23]	WaDI, SWaT, MSL	precision, recall, F1
[24]		accuracy, precision,AUC, FPR
[10]	DS2OS	accuracy, precision, recall, F1, ROC
[45]	DS2OS (distributed smart space orchestration system)	accuracy, precision, recall, F1, FPR, specificity, ROC-AUC
[52]	BaIoT, El Niño	FPR, TPR

**Table A4:** Breakdown of data-sets used.

Data-Set	Application
LANL	cybersecurity
ToN-IoT	IoT/IIoT cybersecurity
HAI 1.0 ICS	system attack
VirusShare	malware analysis
UNB2012	IDS
UNB2018	IDS
UNB2019	IDS
UNSW-NB15	IDS
KDD Cup (1999)	IDS
Bot-IoT	IoT botnet
N-BaIoT	IoT botnet
IoTPOT	IoT DDoS
CMU CERT	insider threat
CERT	insider threat
fraud	fraud detection
yelpChi	fraud detection
ECG	medical imaging
EEG	medical imaging
thyroid	disease classification
PPI	protein interactions
Flickr	image classification
mnist-100	image classification
celeba	image classification (facial)
Reddit	social network
Digg	social network
Bitcoin-alpha	social network
Bitcoin-otc	social network
ACM	citation network
CORA	citation network
KDD 2014	citation network
CiteSeer	citation network
PubMed	citation network
SWaT	IoT soil and water assessment
WaDI	IoT water distribution
MSL	sensor (rover) anomaly
DS2OS traffic	IoT AD
Turbofan	sensor AD (jet engine)
APS Failure Scania Trucks	sensor AD (Trucks)
SECOM	sensor AD (semiconductor construction)
Wafer AD	sensor AD (wafer construction)
El Niño	IoT weather anomaly detection
REALDISP	IoT activity recognition
HAR	IoT activity recognition
DSADS	IoT activity recognition
NAB	IoT time series
SMD	server time series
pwr consumption	time series data
SMAP	soil moisture
news20	text recognition
SIMULATE-3K	dataset generator
Yahoo	multiple
CAIDA 2018	BG traffic
donors	N/A

## Appendix B. Graph-Based Neural Networks

This section highlights the basic structure of GNNs, theories on their usefulness, their uses, and some common NN configurations. All GNNs work on the principal assumption that the data being analyzed by an NN may have hidden information present within it, which, when analyzed by a network designed to view connections between units producing that data (i.e., a graph), may produce more useful insight into a problem. Most GNNs utilize as the input two sets of data: an adjacency matrix *A*, composed of 1’s and 0’s corresponding to edges connecting nodes, and a matrix *X* composed of attributes. If the graphs are directed, *A* only contains a value of 1 in the direction of the edge. For attributes, *X* can contain attribute information for nodes and/or edges, depending on the type of problem being solved by the graph. The GNN itself is composed of blocks, which may repeat, containing graph transformations. Each block performs graph transformations (generally in the form of matrix multiplication), followed by some down-sampling. How these blocks and/or down-sampling techniques work is the basis of all the different types of GNNs available. As can be seen in Figure A1, a basic GNN is nearly identical to a standard MLP, with the main difference being the encoding of the graph matrix information into a large vector. Techniques such as concatenation and flattening of the matrices can be used to generate the vector. Other techniques, such as convolutional graph neural networks (GCNs), do not require this flattening and concatenation, as they are capable of working directly on matrix data.

Most graph-based problems can be broken down into the following categories [66]:

- Node classification, where each node is a component of a system (sensor, basic block of source code, location, person, etc.). The goal is to perform labeling of these nodes to determine their class (what type of sensor a node is, a person’s role in a social network or organization, if a basic block of code contains an error or malware, etc.)
- Node clustering: a regression/prediction technique where nodes in a network are grouped (clustered) together based on similar attributes. The goal is to be able to derive useful information about a graph’s (or network’s) nodes to have an idea of their role without having the aid of labeled classes.
- Edge classification, where an edge is defined as a connection between nodes. Edge classification is used to understand and label connections between nodes. Just how nodes can have attributes explaining something about the node’s identity, edges can also have attributes explaining something about the connection between nodes. Edge classification is used to understand how two nodes interact. An example of this is trying to classify the relationship of two people in a social network.
- Link prediction: a regression technique used to predict whether a pair of nodes will have a connection between them. Link prediction is used in order to predict how nodes may interact in the future. An example of this is a recommender system trying to predict the purchasing habits of a person on a sales website.
- Graph classification: a classification technique where, instead of the individual nodes being classified, the whole system (represented as a graph) is classified. Graph classification is useful when the pertinent information needed is at the higher system/network level, rather than the lower node or edge levels. Examples of graph classification are the determination of the type of network something is or the determination of the function of a particular program. Graph classification can also occur at the sub-graph level. This sub-graph classification can be used to determine unique sub-structures within a graph that may allow for the identification of useful components of a network.
- Graph generation: an encoding technique that is useful for training other neural networks, as well as for producing new information. Graph generation can be used to do things such as increase the amount of data to feed into other GNNs for training or to increase the robustness of adversarial networks and can be used to produce potential new data for practical use (for example, using a graph generator to produce a program).
- Spatial–temporal graph forecasting: a regression technique that takes into account the shifting nature of dynamic graphs in order to predict the future state of a graph. Spatial–temporal graph forecasting is particularly useful when you are dealing with graphs that can add or lose edges and/or nodes, for example the prediction of traffic patterns.
- Graph partitioning: a technique related to node clustering where the goal is to segregate graphs into sub-graphs for the purposes of grouping portions of sub-graphs with interesting properties. This is usually a regression technique, but is related to sub-graph classification.
- Network embedding: a technique used to “graphitize” data for use by GNNs. All of these other problems leverage data represented as a graph; however, GNNs can also be used to generate these graph embeddings. An example of this is taking time series data produced by sensors on a network, running them through a GNN, and outputting an embedding for use in another GNN for node classification.

In order to solve these types of problems, multiple different types of GNNs have been proposed. Each of these GNNs can be categorized into four major categories [66]:

- Recurrent GNNs (RecGNNs);
- Convolutional GNNs (ConvGNNs);
- Graph Autoencoders (GAEs);
- Spatial–Temporal GNNs (STGNNs).

RecGNNs are defined as recurrent, due to their nature of repeating a set of applications over nodes in a graph. As Figure A1 illustrates, the core component of the RecGNN is a repeating (recurring) set of layers, where the output of one layer is fed into the input of another. The goal of this approach is to gain a high-level representation of the nodes in a graph. RecGNNs are some of the first GNNs created to solve graph-based problems. One of the major contributions of RecGNNs is the concept of message passing between nodes. RecGNNs work on the premise that connected nodes will have their node states passed to their neighbors in each successive recurrence block. This allows each node in the graph to learn something about its neighbors (and its neighbors’ neighbors, etc.). This state information can contain further insights into a node’s relationship with the graph at large and can aid in classification and regression problems.

ConvGNNs utilize the concept of convolving information from block to block. How these convolutions are performed depends on the type of ConvGNN used, as well as the overall class of ConvNet. In [66], ConvGNNs were broken down into two categories: spatial and spectral. Spectral ConvGNNs use the concept of noise removal and graph-based signal processing. Spatial ConvGNNs take a page from RecGNNs and utilize the concepts of message passing between nodes to perform their convolutions. Some ConvGNNs (such as GCN [67]) can be modeled as both a spectral and spatial ConvGNN. A key difference between RecGNNs and ConvGNNs is that ConvGNNs usually do not utilize recurrence. Each block in a ConvGNN generally performs with different weighting parameters and/or convolution functions, compared to a RecGNN, which repeats the RecGNN blocks. See Figure A2 for an example of a ConvGNN. ConvGNNs are very popular for their increased efficiency compared to RecGNNs, as well as their increased convenience in combining them with other NN techniques.

The core difference between most ConvGNNs is the actual convolution function. For spectral ConvGNNs, this boils down to how the filter function (used in a convolution) is defined. For spatial ConvGNNs, this boils down to how to combine the state information in the messages from a node’s neighbors and itself. Some popular ConvGNNs are ChebNet [67], GCN [68], GIN [69], GraphSAGE [70], and GAT [71]; each have different convolution functions and have acted as the bases for spin-off ConvGNNs. While spectral models allow different ConvGNNs to be produced by designing new signal filters, spatial ConvGNNs tend to be more general, efficient, and flexible [66].

GNNs at times need to work on very large graphs. In order to do this, some type of down-sampling is generally needed. Just as convolutions are related to CNNs, the commonly used down-sampling technique of pooling (also related to CNNs) is used in GNNs, particularly in ConvGNNs. Some of the most-common pooling techniques used are min–max and average pooling. The purpose of these techniques is to reduce the dimensionality of the embeddings read by the GNN layers in order to avoid things such as over-fitting and computational complexity (which can increase the memory and/or time needed to train GNNs). In addition, pooling techniques can be used to generate graph representations for graph generation problems. This is known as a readout operation and is used by GAEs.

GAEs are particularly useful in generating embeddings for other GNNs or to produce graphs for increased training data or to solve problems such as drug discovery (which is a form of molecular graph generation problem). Comparison between a generated graph and an original (nominal) graph can be used as a type of anomaly detection. GAEs leverage other NN techniques such as multi-layer perceptron (MLP), ConvGNNs, and attention networks (such as GRU and LSTM) in order to generate graph embeddings or produce new graphs. See Figure A3 for an example of a GAE.

Spatial–temporal GNNs (STGNNs) are useful when the network that is being analyzed is dynamic, whether this dynamism is due to the loss of nodes or edges or has a major tie to time series information. Most STGNNs utilize convolutions in order to gain insight into the spatial–temporal nature of the graph. This is performed using either an RNN approach (where some level of historical information from a previous GNN block is used in the calculation of the current GNN block) [72] or a CNN approach (where 1D CNN layers sandwich ConvGNN layers to learn spatial and temporal information) [73]. RNN approaches tend to be more time consuming than CNN approaches [66].

The concept of a neighbor in a GNN is generally defined by how many layers are present in a GNN. Each layer acts as an additional ring of neighbor state information being sent to a node [74]. How this neighborhood information is aggregated is a topic of current study and affects all types of GNNs. In [74], positional encodings and graph modifications to link more nodes together to rely on fewer layers was performed. The goal in [74] was to alleviate the dangers of under-reaching (too few GNN layers, resulting in a failure to pass node state information to distant nodes) and over-squashing (certain nodes acting as bottlenecks for the flow of node information), while avoiding the practice of converting a graph into a fully connected graph, such as as performed in [75,76], which also used positional encodings to retain the original connectivity of the graph, but resulted in the explosion of the computational cost compared to traditional GNN neighbor state information flow.

For a more in-depth analysis of GNNs, please refer to [66].

For the purposes of anomaly detection in the IoT, an IoT network can be seen as a graph G={V,E}, where nodes v∈V are connected in some way by edges $e_{i,j}\in E$. What these edges are can depend on the type of data you are looking at. For instance, if an IoT network comprising sensors that communicate information to one another is used, *E* may consist only of edges between IoT devices that talk to one another. However, for an IoT network where all data are aggregated in a central location, *E* may be born from some spatial component between nodes.

In addition, how the data are formulated can have an impact on the embeddings produced. An IoT dataset comprised of network packet information can produce an embedding very different than an IoT data-set comprised only of the sensor-reported information.

Whether or not graph-based representation of the IoT network is directed or not can be a result of the direction of communication between nodes. For spatial-based IoT network representations, the resultant *G* may be an undirected graph, but for IoT networks that communicate information between nodes, where the information came from may be just as important (if not more important) than assuming a simple undirected edge $e_{i,j}$; therefore, a directed graph may be necessary.

In all of these cases, one can see the inherent benefit of representing sensor networks and some types of data as graphs.
